# A Text Book of Pathological Histology; an Introduction to the Study of Pathological Anatomy

**Published:** 1872-01

**Authors:** 


*A Text Book of Pathological Histology; an Introduction to the Study of Pathological Anatomy.* By Dr. Edward Rindfleisch. Translated from the German by William C. Kloman, M. D., and E. T. Miles, M. D. Philadelphia: Lindsay & Blakiston, 1872.

We cannot undertake to give any just idea of the extent and scope of this work. It is a recent, comprehensive, and we should say, complete treatise upon pathological histology containing the most recent views upon the nature of disease. For the careful student of medicine this work is of inestimable value, while the less ambitious and more practically inclined, will find it too scientific and minute to interest them. It is designed for reading and thinking students of the profession, and will be appreciated by them only.

The minute changes in disease are carefully described, and to add to correct understanding the text is illustrated by two hundred and eight beautifully made wood cuts. Altogether it constitutes one of the most valuable works upon the subject, in our language, and we think will hold first rank as standard in pathological histology.

